# Improvisation – a new strategy in medical education?

**DOI:** 10.3205/zma001337

**Published:** 2020-06-15

**Authors:** Sigrid Harendza

**Affiliations:** 1 Universitätsklinikum Hamburg-Eppendorf, III. Medizinische Klinik, Hamburg, Germany

## Editorial

Suddenly everything is different. A virus keeps the world in suspense and medical education as well. Yet the older teachers among us know quite well what viral diseases are and how they can change undergraduate and postgraduate medical training. During my own undergraduate training the HIV epidemic began, which even after well over 30 years still requires further learning [1], i.e. learning about contents that could not be taught at all the during the time of my studies. The situation is similar with the viral disease hepatitis C. While it was still called hepatitis non-A non-B during my studies [2], it has since been given its current name and everyone, including the teachers, had to learn to understand over the next decades that no vaccination was made possible, but that in the meantime drugs had been developed which can eliminate the hepatitis C virus [3]. Such influences of pathogens on the content of medical studies could be relatively easily dealt with by changing the learning objectives adapted to medical progress.

In case of structural changes, which may become necessary in teaching during epidemics, adaptation processes are not quite so easily implemented. During the EHEC epidemic in 2011, our nephrological-internal ward and several other wards of the University Medical Center Hamburg-Eppendorf accommodated almost exclusively patients suffering from EHEC-induced haemolytic uremic syndrome (HUS) [4]. This is a rare disease of which students only had to have heard of at that time according to the Hamburg Catalogue of Learning Objectives [5]. Within two weeks it was almost impossible for final-year students in internal medicine to see patients with other diseases. Hence, we, as teachers, hat to improvise teaching – in addition to caring for the patients – and maybe the final-year students at that time learned a little less about different diseases but a lot instead about medical behaviour in unknown clinical situations. In the National Competence Based Catalogue of Learning Objectives (NKLM) of 2015, HUS is still listed as a rare disease under point 21.1.58 [http://www.nklm.de, accessed: 04.05.2020], but the labelling with competence level A requires in any case more extensive knowledge than just knowing the name of the disease.

So now, in 2020, it is again a pathogen that influences medical education. But this time the change affects all teachers and all students and almost all teaching and examination structures in medical education and in all other healthcare professions studies as well as in all other courses of study in general – and this worldwide [University World News: https://www.universityworldnews.com/post.php?story=20200324065639773, accessed: 02.05.2020]. For the study of medicine, dentistry, and veterinary medicine, but also for the study of other healthcare professions, we are currently confronted with a rather small-scale structuring of teaching instruction, which prescribes content and form of teaching at most universities right down to the individual lesson. However, in the current situation, which will probably keep us busy for the next few months or even years, the ability to improvise and flexibility are required while keeping the main educational goal in mind. For postgraduate medical education, van Loon and Scheele recently demanded to renounce from detailed regulations and to move towards enabling teachers to engage in curricular innovation that is “only” oriented towards the educational goal [6]. Confidence in the creative design of the curriculum by teachers and their empowerment for free design opportunities [6] should also enable universities to act quickly in times of a pandemic-related lockdown. This kind of action, i.e. the development of one’s own strategies within the frame of one’s own current possibilities paying attention to the global goal, but without prescription of all individual steps in detail, is called mission tactics or command and control with mission in the military. This leadership tactic has proven to be particularly effective during confusing situations to achieve a global goal [7].

The acquisition of improvisation skills is, for example, explicitly required for students of teacher training and is already being practiced in class in some cases [8]. For medical educators and medical students, such techniques of improvisational theatre seem to be useful as well – both for medical activities and for teaching medical students or for designing lessons, respectively [9]. Medical work is unpredictable by its very nature. Medical students must learn to deal adequately with uncertainty inherent in medical problems. This is already being implemented didactically in problem-based learning and leads to a better handling of uncertainty in everyday medical practice [10]. Furthermore, there are frameworks that use techniques of improvisational theatre to enable medical students to learn how to deal with unknown medical situations [11]. These techniques of improvisation could also be appropriate to enable teachers to teach adequately in uncertain times [12]. They appear to be particularly useful for learning communication skills and professional behaviour [13].

But other teaching techniques also help to improvise appropriate medical lessons in times of a lockdown, especially e-learning, of course [14], because it is particularly easy to keep one’s distance. This issue also contains some interesting approaches in scientific work and projects which encourage creative thinking for medical teaching and testing in the current situation, although at the time of their creation there was no mention of SARS-CoV-2 at all. Rauch et al. report on the development of an instructional video for dental students to examine patients with suspected craniomandibular dysfunction [15]. Perhaps a way can be found to allow dental students to practice clinical examination techniques with people in their own homes during a lockdown, guided by such videos. The project could perhaps also be adapted to a 4-step video format based on the so-called Peyton method, as is already used at the Ludwigs-Maximilians-University (LMU) in Munich [https://www.med.moodle.elearning.lmu.de/mod/book/view.php?id=58629&chapterid=1638, accessed: 04.05.2020]. Möltner et al. were able to show that the assessments of student raters in a formative OSCE in general medicine correlated highly with the assessments of medical experts [16]. This scientific finding may also lead to the development of further training and assessment options for medical students as peers for practical and communicative skills. Findyartini et al. were able to show in their study that the motivation profile of medical students is associated with the empathy they express [17]. Thus, this project also offers interesting starting points for teaching and learning empathy. Zimmermann and Kadmon used standardized examinees who had received training for different levels of proficiency in OSCE stations that were filmed and can be used both for quality assurance of OSCE stations and for rater training [18]. This concept can probably be easily and contactlessly used for rater training at other universities. These examples show the essential contribution that projects in medical education research make to enabling teachers to draw on evidence even in times when they have to improvise. So let’s stay tuned to improvised teaching – scientifically sound and creative.

## Competing interests

The author declares that she has no competing interests.
